# Recommendations to minimize tooth root remodeling in patients undergoing maxillary osteotomies

**DOI:** 10.1038/s41598-024-62059-2

**Published:** 2024-06-13

**Authors:** Khalid Ayidh Alqahtani, Reinhilde Jacobs, Oliver Da Costa Senior, Constantinus Politis, Eman Shaheen

**Affiliations:** 1 OMFS IMPATH Research Group, Department of Imaging and Pathology, Faculty of Medicine, KU Leuven, Leuven, Belgium; 2 Department of Oral and Maxillofacial Surgery, University Hospitals Leuven, Leuven, Belgium; 3 Department of Oral and Maxillofacial Surgery and Diagnostic Sciences, College of Dentistry, Sattam Bin Abdulaziz University, Al-Kharj, Saudi Arabia; 4 Department of Dental Medicine, Karolinska Institutet, Stockholm, Sweden

**Keywords:** Root resorption, Le Fort I osteotomy, Orthognathic surgery, SARPE, Orthodontic, Cone-beam computed tomography, Three-dimensional imaging, Teeth root, Diseases, Health care, Risk factors, Signs and symptoms

## Abstract

The purpose of this study was to report root remodeling/resorption percentages of maxillary teeth following the different maxillary osteotomies; i.e. one-piece, two-pieces, three-pieces Le Fort I, surgically assisted rapid palatal expansion (SARPE). The possibility of relationships between root remodeling and various patient- and/or treatment-related factors were further investigated. A total of 110 patients (1075 teeth) who underwent combined orthodontic and orthognathic surgery were studied retrospectively. The sample size was divided into: 30 patients in one-piece Le Fort I group, 30 patients in multi-pieces Le Fort I group, 20 patients in SARPE group and 30 patients in orthodontic group. Preoperative and 1 year postoperative cone beam computed tomography (CBCT) scans were obtained. A validated and automated method for evaluating root remodeling and resorption in three dimensions (3D) was applied. SARPE group showed the highest percentage of root remodeling. Spearman correlation coefficient revealed a positive relationship between maxillary advancement and root remodeling, with more advancement contributing to more root remodeling. On the other hand, the orthodontic group showed a negative correlation with age indicating increased root remodeling in younger patients. Based on the reported results of linear, volumetric and morphological changes of the root after 1 year, clinical recommendations were provided in the form of decision tree flowchart and tables. These recommendations can serve as a valuable resource for surgeons in estimating and managing root remodeling and resorption associated with different maxillary surgical techniques.

## Introduction

Orthognathic surgery, also known as corrective jaw surgery, is a type of surgical treatment that aims to correct and improve the position and function of the jaws and teeth. This surgery is typically indicated for patients with severe skeletal discrepancies or congenital deformities that cannot be corrected with orthodontic treatment alone^1–4^.

One-jaw surgery, also known as single-jaw surgery, is correcting the position and alignment of only one jaw that involves mainly Le Fort I in the upper jaw or bilateral sagittal split osteotomy (BSSO) in the lower jaw. One-jaw surgery is typically recommended for patients with minor jaw discrepancies while bimaxillary surgery, also known as two-jaw surgery involves correcting the position and alignment of both the upper and lower jaws^2,3^.

There are several types of maxillary orthognathic osteotomies that can be performed depending on the patient's individual needs and the severity of their dental and skeletal deficiencies. Most commonly known type of maxillary osteotomy is Le Fort I, which involves cutting and repositioning upper jaw in vertical, horizontal or sagittal directions as a single piece. This technique is indicated for patients with vertical maxillary excess, sleep apnea, midface hypoplasia and/or facial asymmetry^5^. Two-pieces Le Fort I osteotomies are additional osteotomies performed in two separate pieces to correct transversal hypoplasia up until 5-7 mm. Three-pieces Le Fort I osteotomies add the possibility to close an anterior open bite^6–9^. Finally, surgically assisted palatal expansion (SARPE) is another type of maxillary orthognathic osteotomy with gradual expansion of narrow upper jaws or crossbites using a tooth-borne or bone-borne device^10–13^. Whereas multiple-pieces osteotomies need an interposition bone graft, this is not necessary in transverse distraction of the upper jaw with SARPE.

Complications associated with maxillary orthognathic surgery can include nerve damage, infection, bleeding, mouth opening limitation, changes in facial aesthetics and root resorption^14–18^. Additionally, changes in blood flow to the teeth can also be a potential complication during the procedures of maxillary orthognathic surgeries due to the disruption of the blood vessels supplying the teeth leading to a decrease in the delivery of oxygen and nutrients to the teeth causing root remodeling, resorption and even tooth loss^19–21^.

Several methods can be used to evaluate root changes after maxillary orthognathic surgeries such as panoramic radiographs or cone-beam computed tomography (CBCT) scans^22^. These images are utilized to measure the distance between the root apex and certain anatomical landmarks, such as cortical bone or cemento-enamel junction, to determine if root resorption has occurred^12,18^. More appropriately is to use CBCT images to apply 3D analysis allowing more extensive, elaborate and accurate measurements than only linear such as volumetric and morphological changes of the root^23–26^. Three-dimensionally based methods would help surgeons and orthodontist to accurately assess the extent of root resorption/remodeling after maxillary orthognathic surgeries.

The aim of this study is to provide an overview of the potential root resorption/remodeling that can occur following different types of maxillary orthognathic osteotomies and to offer recommendations to surgeons in order to minimize root resorption and provide estimates of root remodeling occurring after various osteotomies.

## Materials and methods

### Ethical approval

This retrospective study was approved by the local Ethical Review Board of the University Hospital Leuven (S57587) and carried out in accordance with the World Medical Association’s Declaration of Helsinki on medical research. Need/requirement for informed consent was waived by ethical committee of the University Hospital Leuven (S57587).

### Patient selection

Inclusion criteria were patients who underwent orthognathic surgery and orthodontic treatment in the University Hospitals of Leuven between ages of 18 and 39 years. Patients who had a previous history of orthognathic surgery, orthodontic treatment, trauma to the maxillofacial region, and syndromic diseases or cleft lip/palate were excluded. In this study, we included 110 patients who met the inclusion criteria. Of these patients, 30 underwent one-piece Le Fort I osteotomy (15 males and 15 females), fifteen underwent two-pieces Le Fort I osteotomy (7 males and 8 females), fifteen underwent three-pieces Le Fort I osteotomy (7 males and 8 females), and twenty underwent SARPE (5 males and 15 females). We also included 30 patients who underwent BSSO and only orthodontic treatment in the upper jaw (15 males and 15 females) that is called orthodontic group. Each of the 110 patients, in accordance with the standard clinical protocol for orthognathic surgery, had two CBCT scans as follows: preoperatively (Pre) and one year postoperatively (1Y).

### CBCT data acquisition

CBCT scans were acquired using the Newtom VGI-evo (Cefla, Imola, Italy) with typical scanning settings of 96–110 kV, 230 × 260–240 × 190 FOV, and 0.2–0.3 mm slice thickness^27^. Following the acquisition of the Pre and 1Y CBCT scans, all data were anonymized and saved in Digital Imaging and Communications in Medicine (DICOM) format.

### Root changes assessment protocol

A previously validated 3D assessment protocol was applied including 3 main steps: segmentation, registration and 3D analysis^28^. Segmentation refers to the process of separating the teeth from the CBCT images, which was performed using a convolutional neural network-based online cloud platform (RELU BV, Leuven, Belgium) called “Virtual Patient Creator” that was previously validated for this purpose^29,30^. The segmented teeth were then saved in a standard Tessellation Language (STL) file format and imported into a fully automated tool within 3-matic software (version 16.0, Materialise N.V., Leuven, Belgium), that selected specific teeth from the upper jaw: central incisors, lateral incisors, canines, first premolars, and second premolars. The tool applied surface-based registration on the crown of one year postoperative tooth to the crown of the preoperative tooth. Additionally, the root part of the teeth was assessed in 3D, including measurements of root length (RL), total root volume (TRV), and volumes for 3 parts of the root: apical third (AP-V), middle third (MP-V), and coronal third (CP-V). To quantify changes over time, 1 year postoperative measurement was divided by the preoperative measurement to obtain volume ratios for remodeling (RE) and length ratios for resorption (RS).

### Patient and surgery factors

In the current study, we investigated the relationships between variables that might be related to root remodeling after maxillary orthognathic osteotomies. Patient related variables are age and gender while treatment related variables included maxillary advancement and planned palatal expansion described by the amount in mm calculated from the number of days the patient used the expander multiplied by the expansion rate of 0.5 mm per day for SARPE patients.

### Statistical analysis

The biostatistician utilized S-Plus 8.0 software for Linux to analyze the data. Mean and standard deviation of percentage of root remodeling (RE) and resorption (RS) in terms of volume, length, and morphological changes (apical part (AP), middle part (MP) and coronal part (CP)) for all teeth and teeth subcategories were reported for the five groups: one-piece Le Fort I, two-pieces Le Fort I, three-pieces Le Fort I, SARPE and orthodontic. The Spearman rank correlation test was used to investigate correlations between gender, age, maxillary advancement (Le Fort I groups), planned palatal expansion (SARPE group), and root remodeling. A p-value of less than 0.05 was used as the threshold for statistical significance.

### Ethical approval

This retrospective study was conducted according to the World Medical Association's Declaration of Helsinki on medical research, and it was authorized by the local Ethical Review Board of the University Hospital Leuven (S57587).

### Patient consent

Need/requirement for informed consent was waived by ethical committee of the University Hospital Leuven (S57587).

## Results

A total of 1075 teeth were evaluated from the 110 patients included in this study. Table 1 shows the clinical characteristics of the participants.

**Table 1:** Clinical characteristics of included participants.

Variable	One-piece	Two-pieces	Three-pieces	SARPE	Orthodontic
Continuous variable, mean ± SD					
Age (years)	26.3 ± 4.8	26.2 ± 3.5	26.7 ± 4.5	25.6 ± 5.5	26.6 ± 4.5
Maxillary advancement (mm)	3.6 ± 1.4	3.35 ± 1.1	3.4 ± 1.3	None	None
Categorical variables, Sample size					
Gender					
Male	15	8	7	9	15
Female	15	8	7	11	15
Teeth	294	156	140	194	291
Teeth subcategories					
Central incisors	60	32	28	40	60
Lateral incisors	60	32	28	40	60
Canines	60	32	28	40	60
1st premolars	57	30	28	37	56
2nd premolars	57	30	28	37	55

### Root changes related to surgery type

Table 2 presents percentage of root resorption and remodeling of the five treatment types for all patients and all teeth. The least percentage of root resorption among groups was orthodontic followed by SARPE, one-piece Le Fort I, two-pieces Le Fort I and three-pieces Le Fort I respectively. In addition, results indicated that SARPE group had the highest percentage of root remodeling, followed by three-pieces Le Fort I , two-pieces Le Fort I, one-piece Le Fort I, and finally the orthodontic group. Root remodeling was the largest in the apical part with a range between 20 ± 0.24% and 29 ± 0.16%.

**Table 2:** Root resorption (RS), remodeling (RE) and morphological changes (*AP* apical part, *MP* middle part, *CP* coronal part) percentages for all teeth following different maxillary osteotomies.

	RS %	RE %	AP %	MP %	CP %
	Mean ± SD	Mean ± SD	Mean ± SD	Mean ± SD	Mean ± SD
Orthodontic	4 ± 0.02	9 ± 0.11	20 ± 0.24	10 ± 0.05	9 ± 0.06
Orthodontic + SARPE	5 ± 0.03	19 ± 0.08	29 ± 0.19	13 ± 0.07	9 ± 0.02
Orthodontic + One-piece Le Fort I	5 ± 0.08	11 ± 0.06	25 ± 0.01	8 ± 0.05	5 ± 0.04
Orthodontic + Two-pieces Le Fort I	6 ± 0.05	12 ± 0.01	26 ± 0.09	10 ± 0.1	6 ± 0.07
Orthodontic + Three-pieces Le Fort I	7 ± 0.06	14 ± 0.09	27 ± 0.04	13 ± 0.03	5 ± 0.01

**Figure 1:**
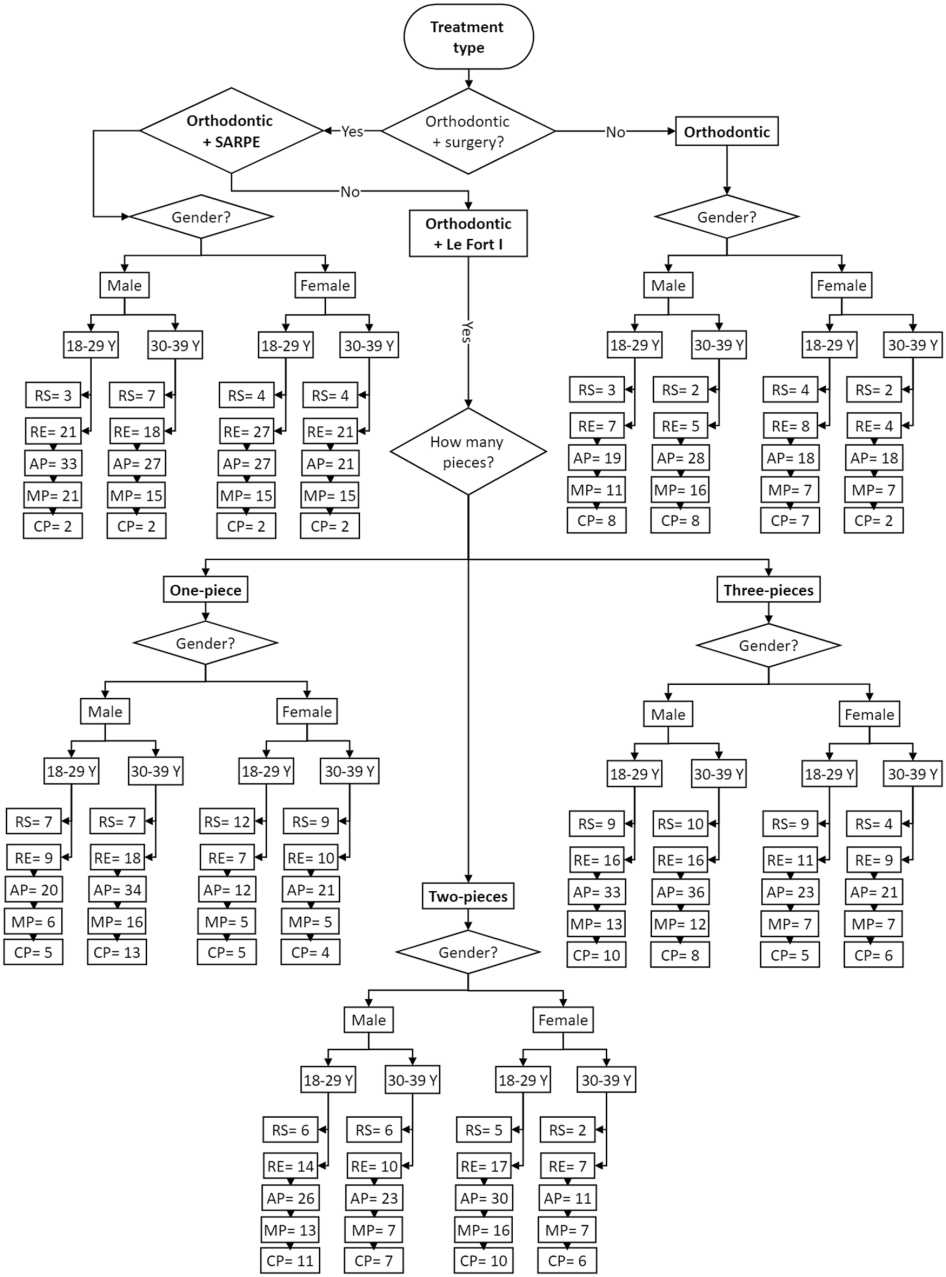
Decision tree flowchart reporting the percentage of root remodeling (RE), resorption (RS) and morphological changes (*AP* apical part, *MP* middle part, *CP* coronal part).

For better visualization, a decision tree flowchart was provided in Fig. 1 that summarizes the percentage of root changes (all five measurements) observed in each group. The flowchart starts by treatment type then divided based on gender then further subdivided into 2 age subgroups: 18–29 years and 30–39 years. For example, a female patient aged between 18 and 29 years who underwent SARPE treatment developed the following: RS: 4%, RE: 27%, AP: 27%, MP: 15% and CP: 2%.

**Figure 2:**
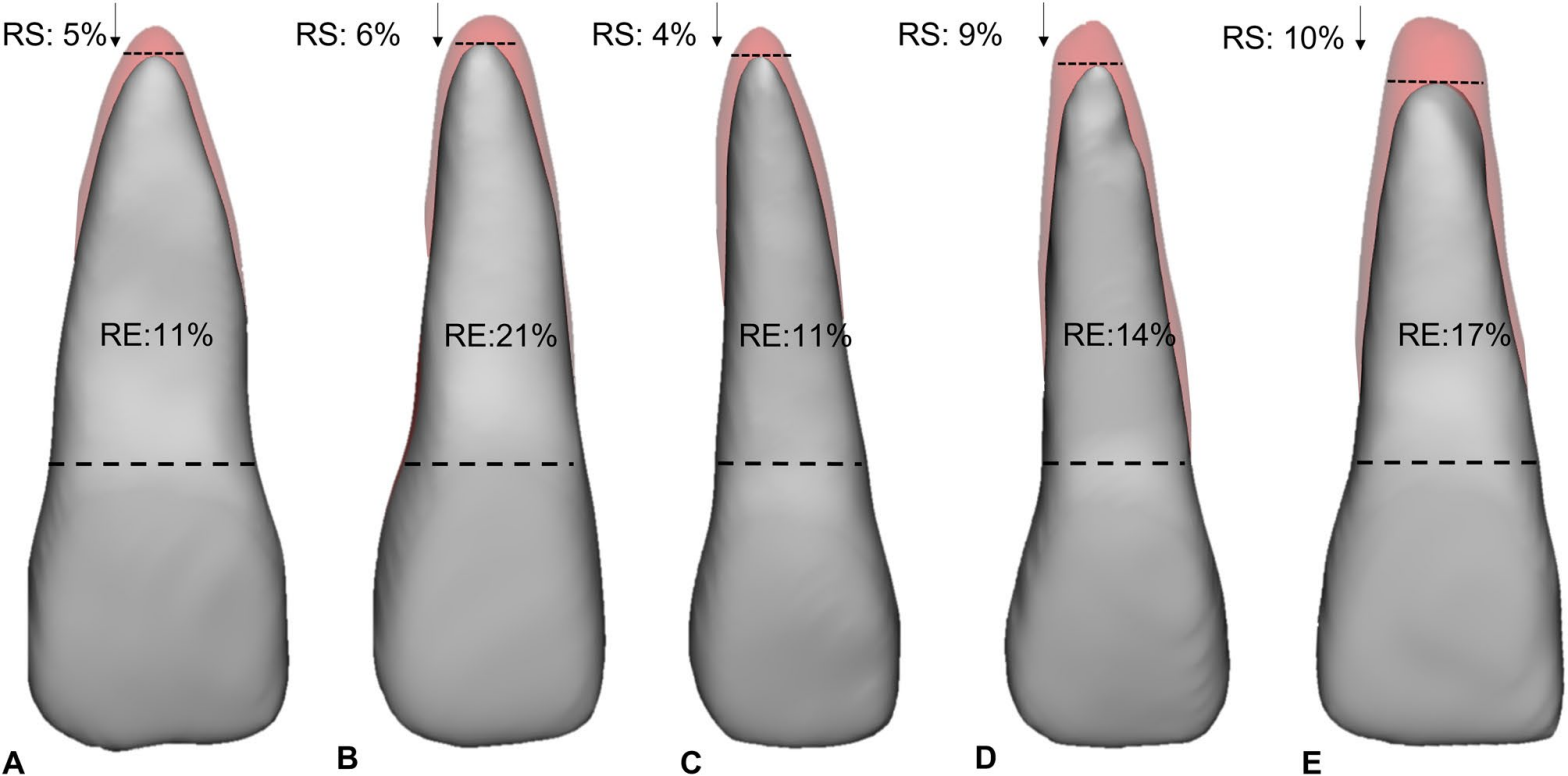
Illustration of root changes in 3D for a typical central incisor of male patients aged between 18–29 years undergoing the five treatment types: (**A**) Orthodontic, (**B**) SARPE + orthodontic, (**C**) One-piece Le Fort I + orthodontic, (**D**) Two-pieces Le Fort I + orthodontic, (**E**) Three-pieces Le Fort I + orthodontic reporting root resorption (RS) and root remodeling (RE). Preoperative tooth is in red transparent and 1 year postoperative tooth in gray.

**Figure 3:**
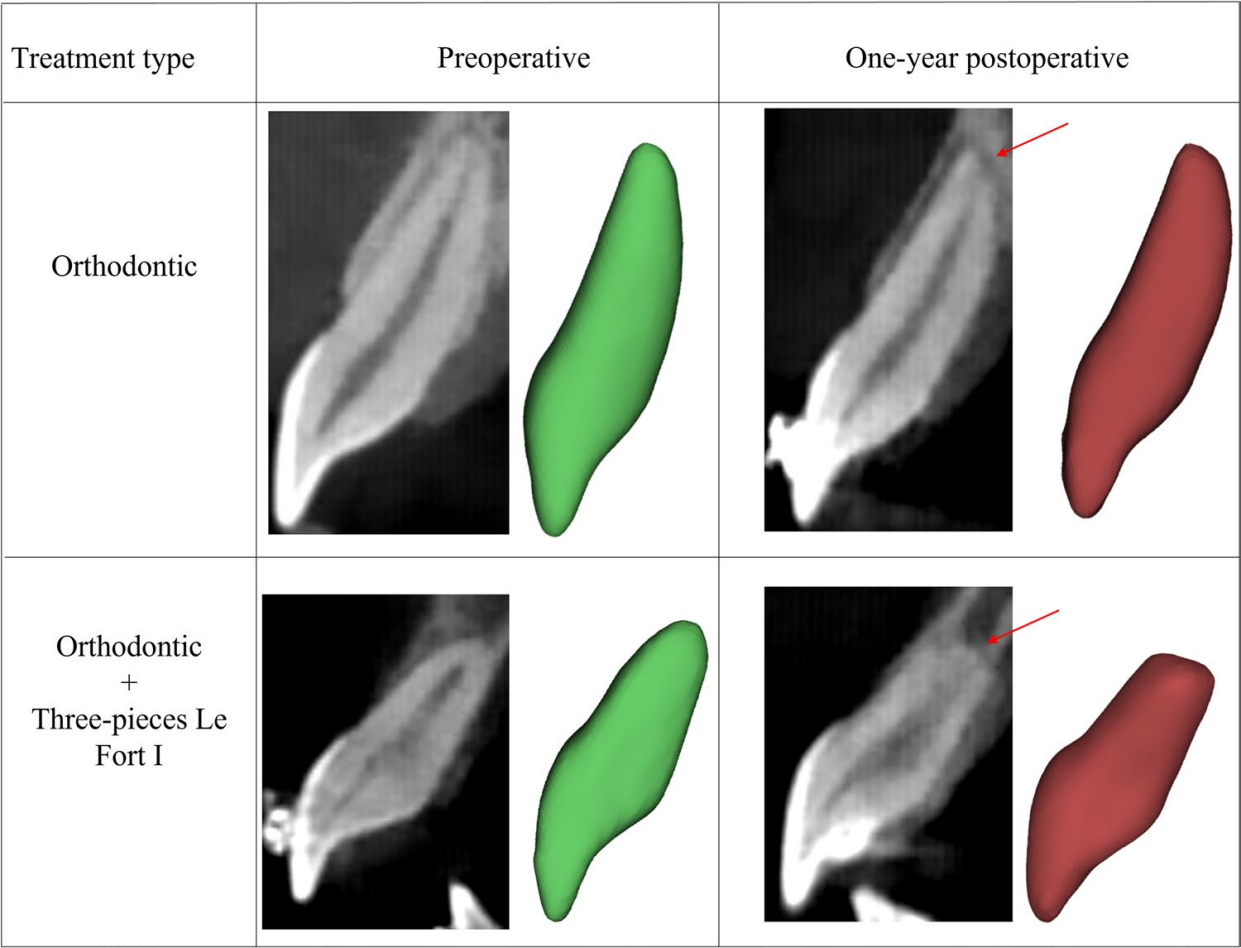
Examples of CBCT images and 3D reconstructions depicting central incisors at preoperative (green) and 1-year postoperative (red) time points for a patient from orthodontic group and another patient from combined orthodontic + three-pieces Le Fort I group, illustrating root changes as indicated by red arrow.

For more detailed results of teeth subcategories, we refer to Table 3 that reported the percentage of RE and RS in terms of volume, length, and morphological changes (AP, MP, CP) for all five treatment groups per gender, age subgroup and tooth subcategory. Figure 2 illustrated via example the root resorption and remodeling measurements for a typical central incisor of a male aged between 18–29 years undergoing the five treatment types investigated in this study as reported in Table 3. Figures 3 showed examples of root changes of central incisors of patients, once undergoing orthodontic treatment and the other undergoing orthodontic combined with three-pieces Le Fort I treatment.

**Table 3:** Results of five measurements: root resorption (RS), remodeling (RE) and morphological changes (*AP* apical part, *MP* middle part, *CP* coronal part) percentages for teeth subcategories divided by treatment type, gender and age subgroups.

Treatment type	Gender	Age (years)	Subcategories	RS %	RE %	AP %	MP %	CP %
Orthodontic	M	18–29	Centrals	5	11	17	9	2
			Laterals	6	12	19	11	8
			Canines	4	8	15	7	5
			1st Premolars	6	16	28	16	11
			2nd Premolars	3	12	17	10	11
	M	30–39	Centrals	9	19	37	16	13
			Laterals	6	18	30	16	18
			Canines	8	16	3	18	16
			1st Premolars	7	20	1	19	20
			2nd Premolars	6	17	1	16	17
	F	18–29	Centrals	7	11	22	8	7
			Laterals	8	12	26	9	8
			Canines	4	6	13	5	4
			1st Premolars	4	8	16	6	7
			2nd Premolars	4	9	13	7	8
	F	30–39	Centrals	4	10	22	7	6
			Laterals	8	13	24	9	9
			Canines	5	11	24	9	7
			1st Premolars	2	8	13	5	8
			2nd Premolars	2	5	6	4	5
Orthodontic + SARPE	M	18–29	Centrals	6	21	24	21	1
			Laterals	3	25	36	23	2
			Canines	4	21	34	20	1
			1st Premolars	1	16	34	19	2
			2nd Premolars	4	22	37	21	2
	M	30–39	Centrals	5	12	14	12	3
			Laterals	4	16	18	14	2
			Canines	5	14	17	14	1
			1st Premolars	9	28	51	22	1
			2nd Premolars	10	21	35	15	3
	F	18–29	Centrals	3	16	24	19	1
			Laterals	4	21	27	19	2
			Canines	4	14	23	14	2
			1st Premolars	4	10	15	7	1
			2nd Premolars	5	18	32	18	3
	F	30–39	Centrals	2	10	11	10	2
			Laterals	2	14	15	13	3
			Canines	4	15	22	14	4
			1st Premolars	8	22	34	19	1
			2nd Premolars	4	16	24	17	2
Orthodontic + One-piece Le Fort I	M	18–29	Centrals	4	11	19	8	3
			Laterals	6	14	26	10	10
			Canines	5	13	23	10	10
			1st Premolars	6	17	33	13	13
			2nd Premolars	6	17	32	14	13
	M	30–39	Centrals	6	9	20	7	6
			Laterals	6	10	18	9	7
			Canines	6	8	16	6	7
			1st Premolars	7	12	32	8	8
			2nd Premolars	7	7	27	6	7
	F	18–29	Centrals	4	14	22	13	11
			Laterals	5	17	26	14	14
			Canines	6	17	31	16	13
			1st Premolars	7	20	38	19	16
			2nd Premolars	5	17	31	16	14
	F	30–39	Centrals	3	11	18	11	9
			Laterals	3	5	8	4	5
			Canines	3	8	16	6	5
			1st Premolars	2	6	15	6	5
			2nd Premolars	0	5	7	3	0
Orthodontic + Two-pieces Le Fort I	M	18–29	Centrals	9	14	33	11	2
			Laterals	12	15	30	9	9
			Canines	5	9	19	8	6
			1st Premolars	7	5	6	4	3
			2nd Premolars	6	3	10	0	0
	M	30–39	Centrals	11	17	33	14	10
			Laterals	17	19	31	16	14
			Canines	11	15	31	14	11
			1st Premolars	16	19	39	16	14
			2nd Premolars	13	20	35	17	15
	F	18–29	Centrals	5	8	14	5	6
			Laterals	7	9	18	7	6
			Canines	3	8	14	7	6
			1st Premolars	2	6	13	5	4
			2nd Premolars	1	2	1	1	1
	F	30–39	Centrals	26	26	61	13	10
			Laterals	12	18	34	11	11
			Canines	3	5	5	0	0
			1st Premolars	0	3	2	1	5
			2nd Premolars	2	4	5	2	5
Orthodontic + Three-pieces Le Fort I	M	18–29	Centrals	10	17	35	13	5
			Laterals	11	17	34	12	9
			Canines	7	12	25	10	8
			1st Premolars	11	20	45	20	13
			2nd Premolars	6	12	24	10	10
	M	30–39	Centrals	7	10	29	6	4
			Laterals	5	17	32	12	12
			Canines	11	18	27	15	12
			1st Premolars	14	15	43	10	11
			2nd Premolars	9	17	32	15	15
	F	18–29	Centrals	12	18	34	13	11
			Laterals	11	14	26	8	8
			Canines	10	8	17	5	6
			1st Premolars	5	7	22	4	5
			2nd Premolars	4	8	15	6	7
	F	30–39	Centrals	4	3	10	1	0
			Laterals	4	8	19	6	5
			Canines	6	11	26	8	9
			1st Premolars	1	16	30	17	10
			2nd Premolars	4	8	18	6	6

### Root remodeling related factors

Regarding patient related factors, the Spearman correlation coefficient showed that younger patients in orthodontic group had a greater chance of developing root remodeling during orthodontic treatment (Spearman correlation = − 0.4, p = 0.01). Among the variables related to surgery, patients with more maxillary advancement were more likely to have root remodeling (Spearman correlation = 0.5, p = 0.018). The other variables evaluated in this study did not reveal statistically significant correlations. In addition, no teeth were lost among the various treatment groups one year postoperatively.

## Discussion

The assessment of root remodeling/resorption after maxillary orthognathic surgeries holds significant importance as it has not been thoroughly investigated in existing literature^9,15,31,32^. By comprehensively evaluating root changes, this study aimed to provide valuable information about the potential effects of orthognathic surgery on the root, which can help optimize treatment outcomes and minimize potential risks.

Previous studies used subjective and linear methods to assess root resorption following either orthodontic treatment or orthognathic surgery^33–38^ however, these methods have limitations in terms of precision and accuracy^39–42^.

Recently, volumetric analysis have emerged as a more reliable and accurate tool for assessing root remodeling after isolated orthodontic treatment^23^. These methods can also aid in quantifying the magnitude and distribution of root remodeling in different dimensions, facilitating a more precise and objective assessment^25,26^. In the present study, a previously validated 3D fully automated protocol for assessing root changes was applied for patients who underwent orthognathic surgery^28^.

Root resorption is a common complication that may occur during isolated orthodontic treatment or combined with orthognathic surgery. In this study, the overall percentage of the amount of root resorption among different treatment types ranged between 4 and 7% and can be considered minimal. On the other hand, results have shown that SARPE group is associated with the highest percentage of root remodeling described by root volume measurements. A possible explanation can be that SARPE is often performed in patients with transverse maxillary deficiency or a constricted maxillary arch, where roots of teeth are already positioned close to cortical bone, potentially leading to root remodeling because of increased mechanical forces. In addition, tooth-borne appliances may have more impact on root remodeling since they transmit more force directly to teeth, which can lead to increased pressure on the periodontium and root surfaces^11,12^. This pressure can cause cellular and molecular changes in the periodontium and alveolar bone, leading to remodeling of surrounding tissues and possible root resorption^22^. In our study, all patients undergoing SARPE were treated with a tooth-borne rapid palatal expansion appliance with an expansion rate of 0.25 mm twice a day. However, it is important to note that even with a slower rate of expansion, there is still a risk of root remodeling in patients undergoing SARPE.

Three-pieces Le Fort I surgery also resulted in root remodeling followed by two-pieces and one-piece Le Fort I surgery respectively, due to the involvement of more segments, potentially larger surgical movements and blood flow impairment^6,16,43^. On the other hand, the orthodontic group had the least amount of root remodeling. The extent and pattern of root changes can vary depending on the type and magnitude of surgical movements, treatment sequence, and patient-specific factors. Therefore, careful consideration of the specific treatment combination and its order is crucial in treatment planning and postoperative management to minimize the risk of root resorption or remodeling and optimize patient outcomes.

In this study, a negative correlation has been observed between root remodeling and younger patients in the orthodontic group. There are several possible reasons for this negative correlation. First, younger patients generally have less mature root structures, as root development continues until late adolescence or early adulthood. Immature roots may be more susceptible to remodeling or resorption in response to the mechanical forces applied during orthodontic treatment or orthognathic surgery. Additionally, younger patients may have more active cellular processes in the periodontal ligament and bone, which could potentially influence root remodeling^43^.

In contrast, a positive correlation has been observed between root remodeling and the amount of maxillary advancement. The relationship between increased maxillary advancement during orthognathic surgery and increased root remodeling can be attributed to the repositioning of the maxilla during surgery contributing to changes in blood flow and positioning. This can lead to altered forces on the roots, including increased tensile and compressive forces on the labial and palatal surfaces of the upper teeth, as well as shear forces due to changes in tooth movements^7^.

According to the findings of this study, the amount of root resorption was considered minimal within the range of 4% to 7% for isolated orthodontic treatment or combined with maxillary surgery, respectively. In case of diagnosed root resorption, caution should be taken when planning SARPE or large maxillary advancement for Le Fort I osteotomies as they were associated with increased root remodeling.

On the other hand, in patients with orthodontic relapse resulting in an anterior open bite and narrow maxilla, a three pieces Le Fort I osteotomy is preferred over a SARPE procedure followed by a single piece Le Fort I osteotomy. Furthermore, estimation of root resorption/remodeling for each treatment type, gender, age group and tooth subcategories were presented in Tables 2 and 3 to assist surgeons with decision making of treatment planning.

The study has limitations that should be considered in interpreting the findings. Firstly, the follow-up duration of only one year may not capture the complete remodeling or resorption processes that can occur over a longer timeframe. Secondly, the study relied on the planned amount of palatal expansion (SARPE group) as the only available documented measurement of expansion in the patient files, which may not accurately reflect the actual amount of expansion achieved during the surgical procedure. Further studies should be conducted on prospectively controlled trials with a larger sample size. The findings of more elaborated studies may allow generating a larger amount of data to build a predictive model for assessing root resorption risk. This might allow surgeons and orthodontist to more effectively predict outcomes and tailor treatments to individual patients, ultimately leading to improved surgical outcome with less complications.

## Conclusions

The present study is the first ever to address root remodeling and resorption 1 year after a combined orthodontic/orthognathic treatment procedure, meanwhile looking to volumetric, linear, and morphological changes and compare these to root remodeling occurring after isolated orthodontic treatment. The current recommendations give more insight to surgeons in estimating possible root remodeling and resorption associated with different maxillary surgery techniques serving a valuable resource for patient specific treatment planning.

## Data Availability

The data that support the findings of this study are available from the corresponding author upon reasonable reason.
